# The study of blood transcriptome profiles in Holstein cows with miscarriage during peri-implantation

**DOI:** 10.5713/ajas.17.0793

**Published:** 2018-05-31

**Authors:** Guoli Zhao, Yanyan Li, Xiaolong Kang, Liang Huang, Peng Li, Jinghang Zhou, Yuangang Shi

**Affiliations:** 1 Department of Animal Husbandry, Agricultural College of Ningxia University, Yinchuan, Ningxia 75004, China; 2 Helan Mountain Diary Company of Ningxia, Yinchuan, Ningxia 75004, China

**Keywords:** Differentially Expressed Genes, Miscarriage, Pregnancy, Progesterone, RNA Sequencing

## Abstract

**Objective:**

In this study, the transcriptome profile of cow experiencing miscarriage during peri-implantation was investigated.

**Methods:**

Total transcriptomes were checked by RNA sequencing, and the analyzed by bioinformatics methods, the differentially expressed genes (DEGs) were analysed with hierarchical clustering and Kyoto encyclopedia of genes and genomes (KEGG) pathway analysis.

**Results:**

The results suggested that serum progesterone levels were significantly decreased in cows that miscarried as compared to the pregnant cows at 18, 21, 33, 39, and 51 days after artificial insemination. The RNA sequencing results suggested that 32, 176, 5, 10, and 2 DEGs were identified in the pregnant cows and miscarried cows at 18, 21, 33, 39, and 51 d after artificial insemination. And 15, 101, 1, 2, and 2 DEGs were upregulated, and 17, 74, 4, and 8 DEGs were downregulated in the cows in the pregnant and miscarriage groups, respectively at 18, 21, 33, and 39, but no gene was downregulated at 51 d after artificial insemination. These DEGs were distributed to 13, 20, 3, 6, and 20 pathways, and some pathway essential for pregnancy, such as cell adhesion molecules, tumor necrosis factor signaling pathway and PI3K-Akt signaling pathway.

**Conclusion:**

This analysis has identified several genes and related pathways crucial for pregnancy and miscarriage in cows, as well as these genes supply molecular markers to predict the miscarriage in cows.

## INTRODUCTION

For a successful pregnancy, the blastocyst’s implantation to the maternal endometrium is crucial [1]. The rate of miscarriage caused by embryonic and fetal losses is approximately 40% in cattle [2–4]. Previous study suggested that many genes and signalling pathway are involved in early pregnancy, and some genes are essential for maintaining pregnancy, and the miscarriage was triggered by the dysfunction of these genes, such as cadherin 2 (*CDH2*), which is a gene of cell adhesion molecules (CAMs) [5]; collagen type VI alpha 3 chain (*COL6A3*), thrombospondin 1 (*THBS1*), fibronectin 1, integrin beta 4 (*ITGB4*) [6], which are all PI3K-Akt signaling pathway genes; matrix metallopeptidase 14 (*MMP14*), vascular cell adhesion molecule 1 (*VCAM1*) [7], matrix metallopeptidase 9 (*MMP9*), which are all tumor necrosis factor (TNF) signaling pathway genes; additionally, MAPK signaling pathway [8] and Hippo signaling pathway [9] are involved in maintaining pregnancy. In addition, miscarriage was caused by infectious diseases such as bovine herpesvirus 1 and brucellosis [10,11]. The miscarriage event eventually induces damages in the pregnancy mechanism of the cow. For this reason, studying the prevention and diagnosis of bovine miscarriages greatly attracted researchers in the past.

The steroid hormone progesterone (P_4_) is essential for the establishment and maintenance of pregnancy [12]. Elevated concentrations of P_4_ during the post-conception period generally result in higher pregnancy rate and greater conceptus survival [13,14]. Therefore, the concentration of P_4_ can be used as a marker for determining the early stages of pregnancy.

Elevated concentrations of circulating P_4_ have been associated with an advancement of conceptus elongation, and an elongated conceptus is immediately implanted to endometrial epithelial cells in cattle between 18 and 19 d [15]. Therefore, the endometrium and the embryos of pregnant cows at 18 to 19 d were used to detect the related genes involved in the pregnancy. However, it is difficult to collect endometrium and embryo samples in cows, which adds to the difficulty in diagnosing miscarriage and pregnancy. Blood serum can provide an optimal sample to explore genes involved in pregnancy and miscarriage. Thus, we aimed to study the transcriptome of blood serum samples of both miscarried and pregnant cows, through RNA sequencing, which could help us better understand any novel marker genes responsible for diagnosing pregnancy and miscarriage.

## MATERIALS AND METHODS

### Selection of cows

Primiparous holstein cows aged 12 to 18 months were selected for this study. These cows without history of reproductive ailments or infectious diseases, were naturally coming in heat and brought for artificial insemination (AI). All animals were provided with balanced ration and *ad libitum* water throughout the day. Physiological responses were examined regularly to ensure healthy status of the cows. Animal pregnancy was ensured by checking of nonreturn of heat and progesterone level (through ELISA test), whereas miscarriage was confirmed through the examination of embryonic and endometrium dysfunctions/symptoms. All procedures regarding animal care and treatments were approved by the Committee for Experimental Animals of Ningxia University.

### Collection of blood

The blood of pregnant and miscarriaged cows at 18 d, 21st, 33rd, 39 d and 51st post-AI was collected according to previous study [16], at least 6 cows were used to collect blood in each group. Each of the blood samples, after collection, was centrifuged for 10 min at 3,000 rpm and separated into 3 layers. The blood serum and leukocytes residing in the upper and middle layers, respectively, were collected gently by RNase-free tips. For leukocytes, an additional Trizol reagent (Takara, Dalian, China) was also added with it in a 1.5-mL RNase-free centrifuge tube. The blood serum, leukocytes were preserved as liquid for progesterone estimation and RNA sequencing, and polymerase chain reaction (PCR).

### RNA extraction, RNA sequencing, real-time PCR and ELISA

Total RNA was extracted using Trizol reagent according to the manufacturer’s instructions. The quality of RNA was observed with a nano-drop 2000 spectrophotometer (RNA concentration and purity) and DNA agarose gel (RNA integrity). Finally, those of good RNA samples were used for sequencing and real-time PCR.

The RNA sequencing was performed by Novogene Company (Beijing, China) following standard protocols.

For PCR analysis, the DNA was reverse-transcribed using PrimeScript RT reagent kit (Takara, China) according to the manufacturer’s instructions. The real-time PCR procedure was performed with ABI7500 Fast, and the data were analysed by ABI7500 Fast software. A 10 μL SYBR Premix Ex Taq II was used for detection of PCR product from a 20 μL volume mixture that also included 2.0 μL complementary DNA (≈ 25 ng total RNA), 0.8 μL of each primer at 10 mM, 0.4 μL ROX reference dye, and 6 μL of nuclease-free water. The PCR run included a 1 cycle run at 95°C for 30 s, a 40 cycles run at 95°C for 5 s, which followed by another 40 cycles run at 60°C for 30 s.

Gene mRNA quantification was performed by the 2 ^−ΔΔCt^ method according to previous study [17], and glyceraldehyde-3-phosphate dehydrogenase was used as a reference gene to normalize the target gene expression. The primer of genes was shown in Table 1.

The blood serum concentration of progesterone was measured by an ELISA kit following manufacturer’s protocol at the Beijing Sino-uk Institute of Biological Technology with an ELISA kit according to the manufacturer’s instructions.

### Statistical analysis

Experiments on each group of animals were replicated (3 times or more) for more accurate estimation. Data obtained in the study were analyzed with GraphPad Prism 5 using paired *t*-tests. Significant difference was defined as p<0.05.

## RESULTS

### Progesterone levels

**Figure 1:**
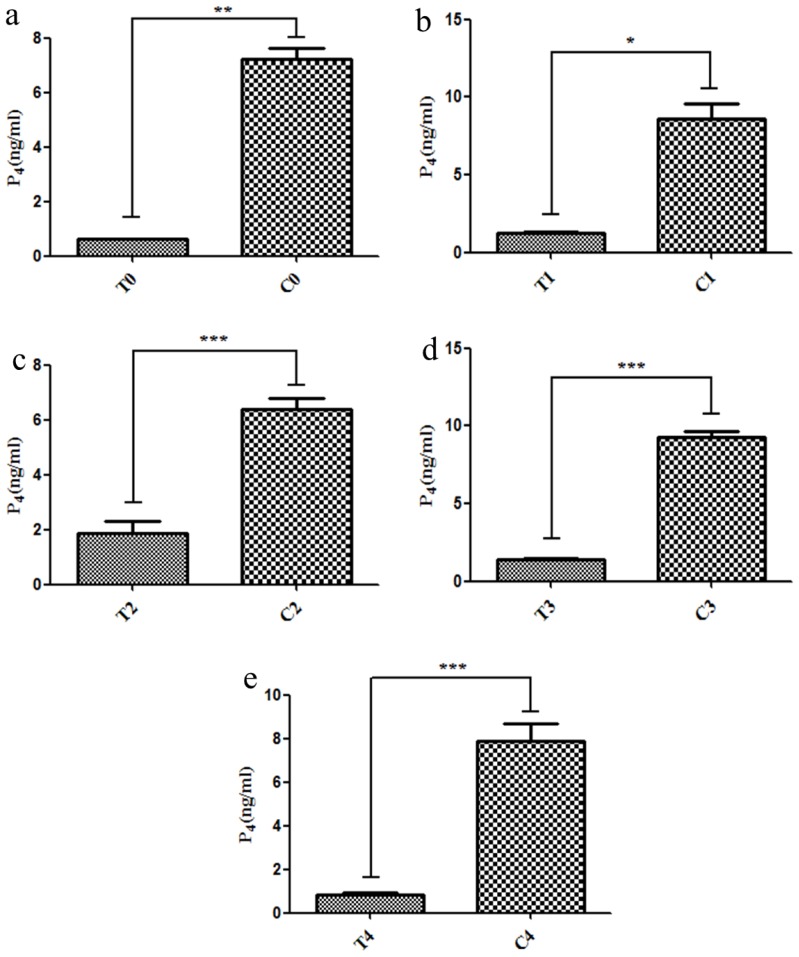
The progesterone levels in pregnant cows and cows who miscarried. The progesterone (P_4_) levels were significantly decreased in the miscarriage cow group than in the pregnant cow group at 18 d (a), 21 d (b), 33 d (c), 39 d (d), and 51 d (e). T, miscarriage cow group; C, pregnant cow group. * p<0.05, ** p<0.01, *** p<0.001.

Figure 1(a) to 1(e) showed that the progesterone concentration was significantly lower in cows who miscarried than in pregnant cows at 18 d post-AI (Figure 1a; p<0.01), at 21 d post-AI (Figure 1b; p<0.05), at 33 d (Figure 1c; p<0.001), at 39 d (Figure 1d; p<0.001) and at 51 d (Figure 1e; p<0.001). In addition, the pregnancy and miscarriage were further confirmed by B-ultrasonic diagnosis (data not shown).

### Differentially expressed genes in cows

#### RNA sequencing in cows on 18th post-AI

**Figure 2:**
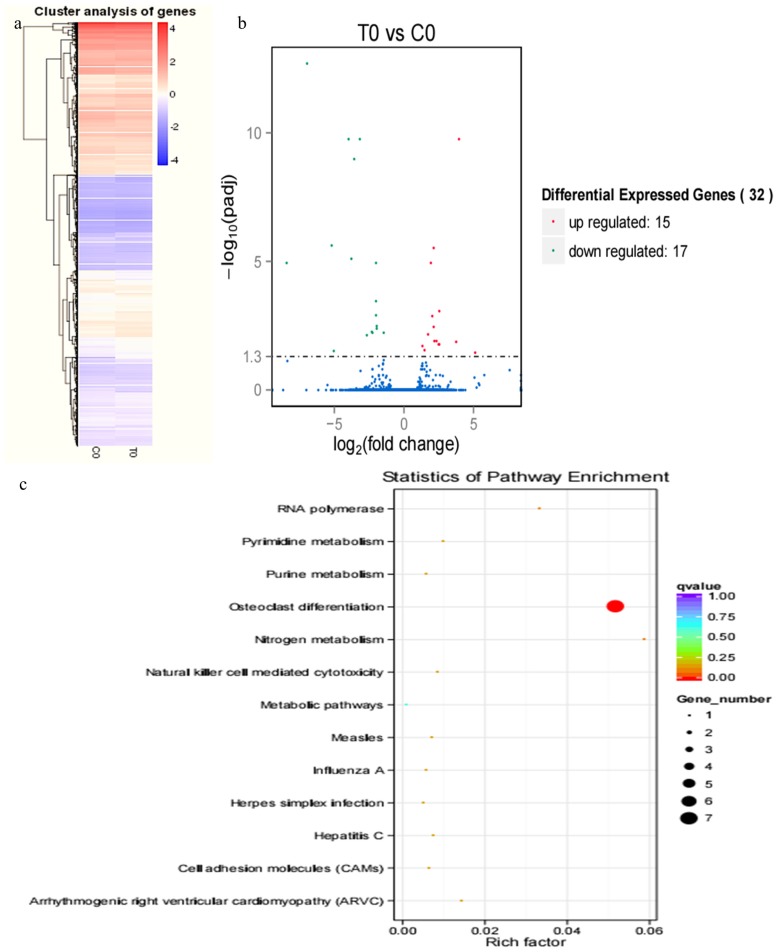
RNA sequencing results in pregnant and miscarriage cow groups at 18 d post-artificial insemination. Hierarchical clustering of differentially expressed genes (DEGs) showed that DEGs at 18 d were different between the pregnant cows and the cows who miscarried cows. (b) DEGs were screened in a volcano plot, and results suggested that 32 DEGs were found; 15 were upregulated and 17 were downregulated. (c) Kyoto encyclopedia of genes and genomes (KEGG) pathway analysis showed that DEGs were distributed in 13 pathways.

Hierarchical clustering of differentially expressed genes (DEGs) showed that DEG expression patterns on 18th were different between the pregnant and miscarriage cows (Figure 2a). DEGs were screened in a volcano plot, and results suggested that 32 DEGs were found; 15 were upregulated and 17 were downregulated (Figure 2b). Kyoto encyclopedia of genes and genomes (KEGG) pathway analysis showed that DEGs were distributed in 13 pathways (Figure 2c). In addition, the expression of two DEGs, leukocyte immunoglobulin like receptor A4 (LILRA4) which belongs to natural killer cell mediated cytotoxicity pathway, and CDH2 belongs to cell adhesion molecules pathway, two genes are all closely correlated with pregnancy, was confirmed by real-time PCR, a finding consistent with the RNA sequencing results. These two genes were significantly downregulated in the miscarriage cow group than in the pregnant cow group (Figure 7a, b; p<0.05).

#### RNA-sequencing in cows on 21st post-AI

**Figure 3:**
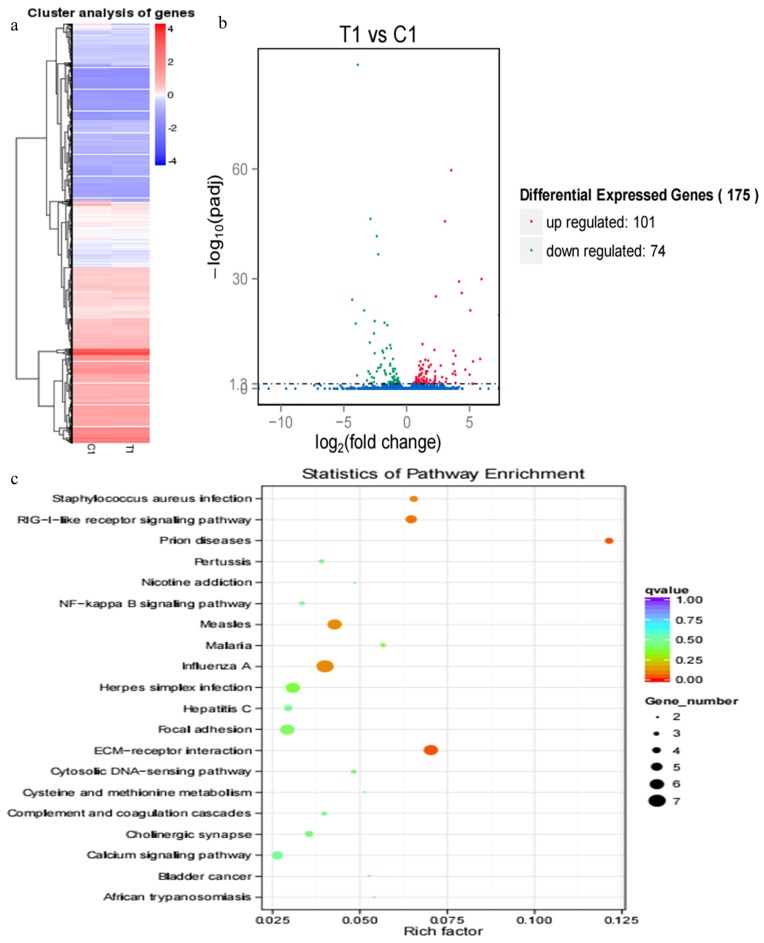
RNA sequencing results of pregnant cows and cows who miscarried at 21 d post-artificial insemination. (a) Hierarchical clustering of differentially expressed genes (DEGs) showed that DEGs at 21 d were different between the pregnant and miscarriage cows. (b) DEGs were screened in a volcano plot, and results suggested that 175 DEGs were found; 101 were upregulated and 74 were downregulated. (c) Kyoto encyclopedia of genes and genomes (KEGG) pathway analysis showed that DEGs were distributed in 20 pathways.

Hierarchical clustering of DEGs showed that DEG expression patterns in 21 d were different between the pregnant and miscarriage cow groups (Figure 3a). DEGs were screened in a volcano plot, and results suggested that 175 DEGs were found; 101 were upregulated and 74 were downregulated (Figure 3b). KEGG pathway analysis showed that DEGs were distributed in 20 pathways (Figure 3c). The expression of two DEGs, MMP14 and VCAM1 belong to TNF signaling pathway, which are closely correlated with pregnancy, was confirmed by real-time PCR, a finding consistent with the RNA sequencing results, the two genes closely correlated with pregnancy, MMP14 and VCAM1, were significantly upregulated in the miscarriage cow group than in the pregnant cow group (Figure 7c, d; p<0.05).

#### RNA sequencing in cows on 33rd, 39th, and 51st post-AI

**Figure 4:**
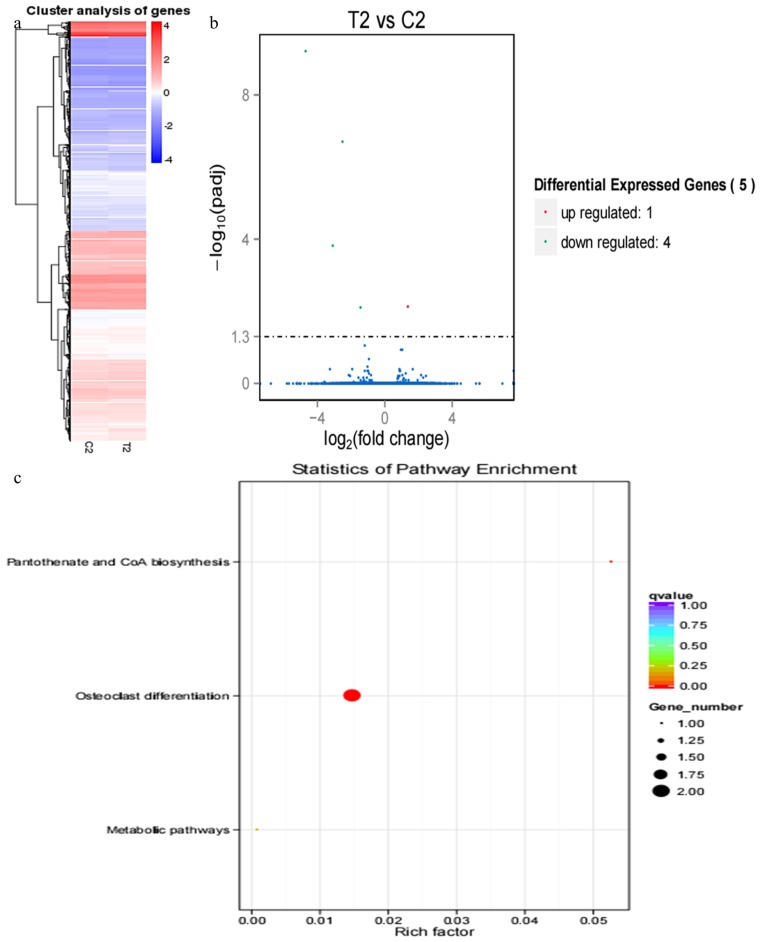
RNA sequencing results of pregnant cows and cows who miscarried at 33 d post-artificial insemination. (a) Hierarchical clustering of differentially expressed genes (DEGs) showed that DEGs at 33 d were weakly different between the pregnant cows and cows who miscarried. (b) DEGs were screened in a volcano plot, and results suggested that five DEGs were found; one was upregulated and four were downregulated. (c) Kyoto encyclopedia of genes and genomes (KEGG) pathway analysis showed that DEGs were distributed in three pathways.

**Figure 5:**
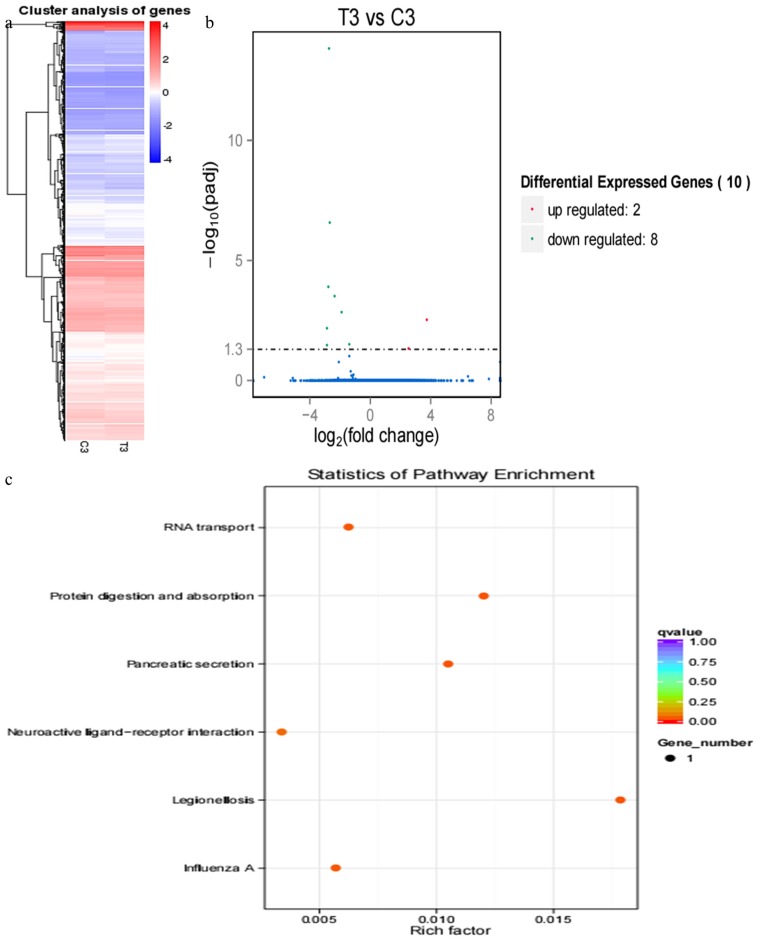
RNA sequencing results of pregnant cows and cows who miscarried at 39 d post-artificial insemination. (a) Hierarchical clustering of differentially expressed genes (DEGs) showed that DEGs at 39 d were weakly different between the pregnant cows and the cows who miscarried. (b) DEGs were screened in a volcano plot, and results suggested that 10 DEGs were found; 2 were upregulated and 8 were downregulated. (c) Kyoto Encyclopedia of Genes and Genomes (KEGG) pathway analysis showed that DEGs were distributed in six pathways.

**Figure 6:**
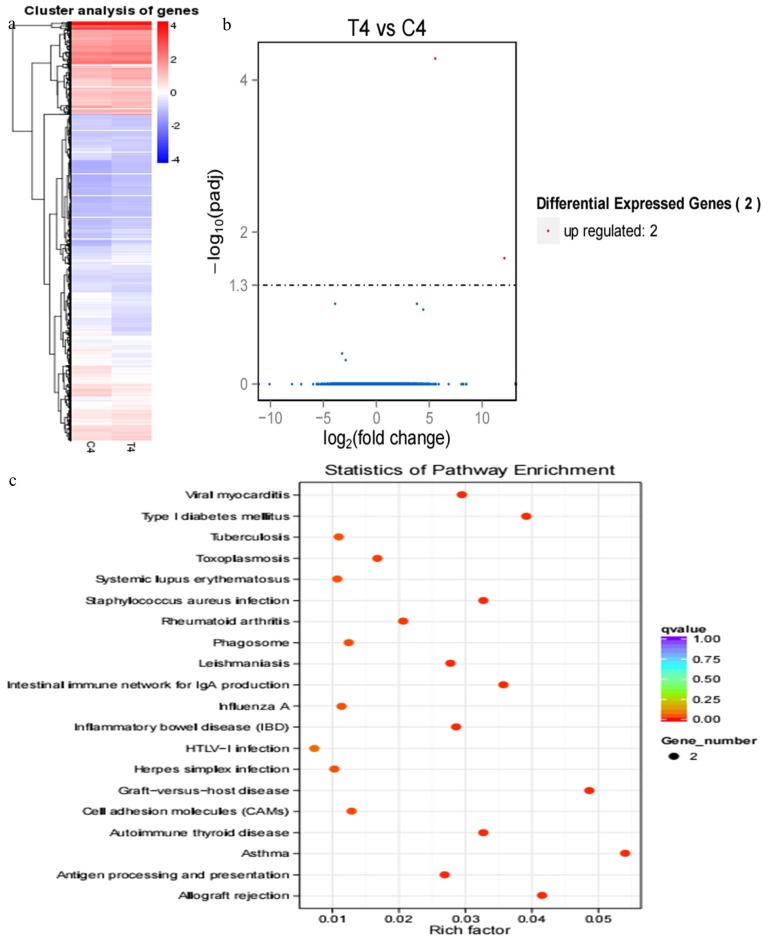
RNA sequencing results of pregnant and miscarriage cows at 51 d post-artificial insemination Hierarchical clustering of differentially expressed genes (DEGs) showed that DEGs at 51 d were weakly different between the pregnant cows and the cows who miscarried. (b) DEGs were screened in a volcano plot, and results suggested that two DEGs were found; two were upregulated and none were downregulated. (c) Kyoto Encyclopedia of Genes and Genomes (KEGG) pathway analysis showed that DEGs were distributed in 20 pathways.

**Figure 7:**
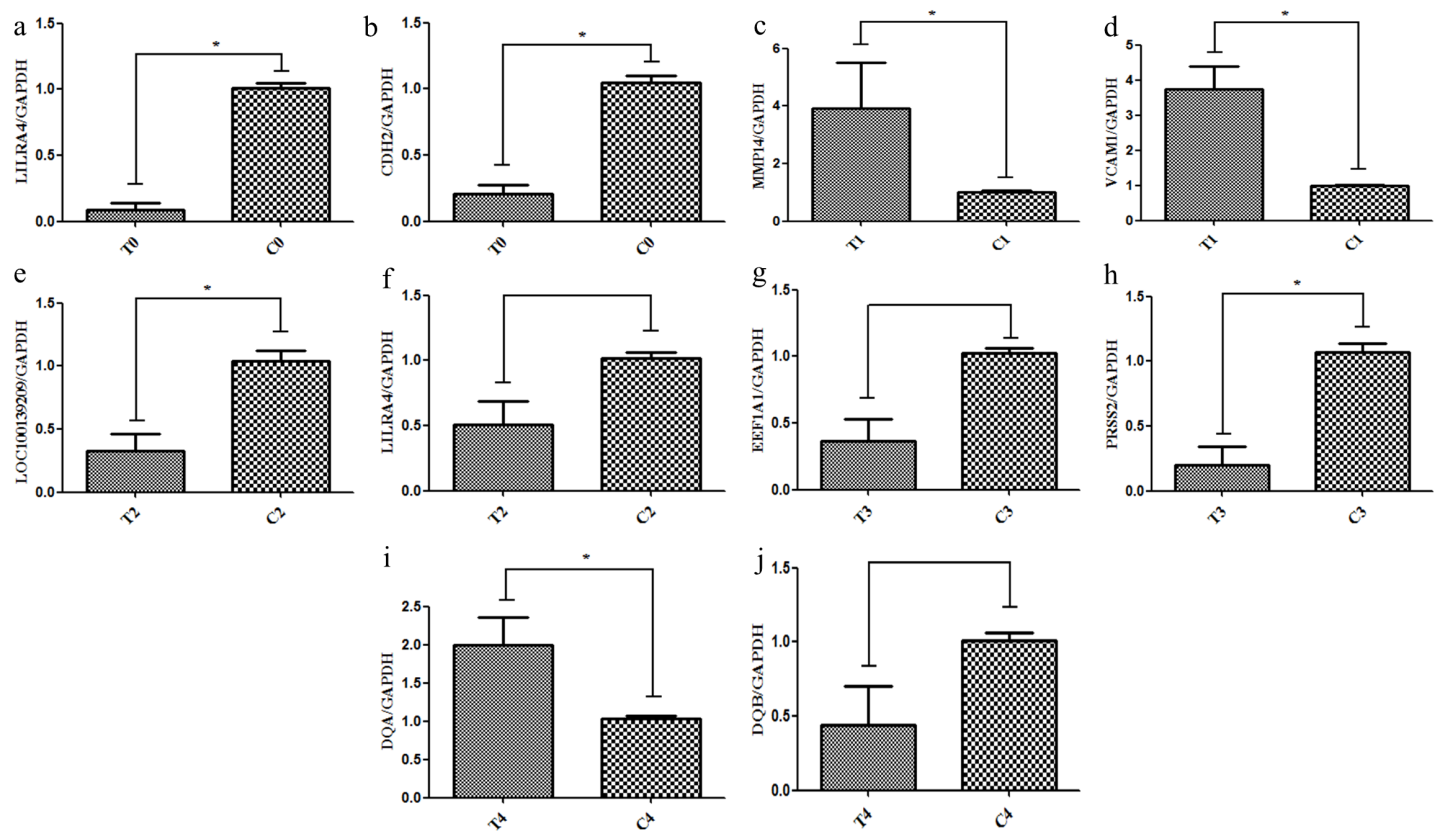
The differentially expressed genes (DEGs) were confirmed by real-time polymerase chain reaction (PCR). Two DEGs of *LILRA4* (a) and *CDH2* (b) at 18 d, *MMP14* (c) and *VCAM1* (d) at 21 d, *LOC100139209* (e) and *LILRA4* (f) at 33 d, *EEF1A1* (g) and *PRSS2* (h) at 39 d, and *BoLA-DQA* (i) and *BoLA-DQB* (j) at 51 d post-artificial insemination (AI) was further confirmed by real-time PCR. *LILRA4*, leukocyte immunoglobulin like receptor A4; *CDH2*, cadherin 2; *MMP14*, matrix metallopeptidase 14; *VCAM1*, vascular cell adhesion molecule 1; *LOC100139209*, leukocyte immunoglobulin-like receptor subfamily A member 6; *EEF1A1*, eukaryotic translation elongation factor 1 alpha 1; *PRSS2*, serine protease 2; *BoLA-DQA*, Bos taurus MHC class II antigen A. * p<0.05, ** p<0.01, *** p<0.001.

Compared with results of hierarchical clustering of DEGs at 18 and 21 d, hierarchical clustering of DEGs showed that DEG expression patterns at 33 d (Figure 4a), 39 d (Figure 5a), and 51 d (Figure 6a) were weakly different between the pregnant and miscarriage cow groups. Volcano plot results suggested that 5 DEGs were found at 33 d; 1 was upregulated and 4 were downregulated (Figure 4b), KEGG pathway analysis showed that DEGs were distributed in 3 pathways (Figure 4c); 10 DEGs were found at 39 d, 2 were upregulated, and 8 were downregulated (Figure 5b). KEGG pathway analysis showed that DEGs were distributed in 6 pathways (Figure 5c); 2 DEGs were found at 51 d, 2 were upregulated, and no DEG was downregulated (Figure 6b). KEGG pathway analysis showed that DEGs were involved in 20 pathways (Figure 6c). To further confirm the results of RNA sequencing, two genes, namely *LOC100139209* (leukocyte immunoglobulin-like receptor subfamily A member 6) and *LILRA4* at 33 d (Figure 7e, 7f), eukaryotic translation elongation factor 1 alpha 1 and *PRSS2* (serine protease 2) at 39 d (Figure 7g, 7h), and *Bos taurus* MHC class II antigen A (*BoLA-DQA*) and *BoLA-DQB* (Figure 7i, 7j) were checked by real-time PCR, and results suggested that only the expression of BoLA-DQB was not consistent with the results of RNA sequencing.

## DISCUSSION

Approximately 50% of miscarriages occur during the process of embryo implantation, when blastocyst hatching and embryo attachment occur in the endometrium of cattle [18]; the establishment of proper maternal–fetal communication and optimal uterine environment are essential. Although various studies were implemented to explore the expression of transcripts and proteins in bovine endometria and conceptus [19–21], the mechanism of pregnancy and miscarriage was not fully elucidated.

Our results revealed that the progesterone concentration in cows who miscarried was remarkably decreased compared with that during pregnancy. In mammals, including cows, the continued P_4_ secretion is indispensable for the establishment and maintenance of pregnancy [22]. In this regard, a report by Psychoyos [23], stated that various genes expressions that were directly or indirectly regulated by P4 in endometrium were involved with the regulation of numerous uterine functions for normal embryonic development through endometrial secretions. Therefore, elevated progesterone concentration maintains the pregnancy, and the miscarriage event in the cow was triggered by decreased progesterone level.

Our studied serum transcriptome profiles in cows provided a greater insight into the function of various DEGs at different pregnancy periods, and results suggested that several DEGs were identified in pregnant cows and miscarried cows at 18 and 21 d, but only a few DEGs were identified at 33, 39, and 51 d, and this might be attributed to the embryo implantation occurred on the 18 to 19 d post-AI [15], and 50% of miscarriages in cows occur during the process of embryo implantation [18]; indeed, the embryo implantation was regulated by many embryonic and endometrium genes. At 18 d, 32 DEGs involved in pregnancy and miscarriage were identified, such as LILRA4, LILRA6, leukocyte immunoglobulin-like receptor subfamily B member 3, leukocyte immunoglobulin-like receptor subfamily A member 6, DNA-directed RNA polymerase I subunit RPA12, CDH2, natural cytotoxicity triggering receptor 1, *Bos taurus* 2′,5′-oligoadenylate synthetase 1 (OAS1Y, 40/46kDa), and carbonic anhydrase 5A; in addition, the importance of CDH2 on embryo implantation was emphasized by previous study [24]. Furthermore, 175 DEGs were identified at 21 d, and those genes are mainly distributed in 20 pathways, such as PI3K-Akt signalling pathway, tumour necrosis factor signalling pathway, MAPK signalling pathway, and Hippo signalling pathway, COL6A3, THBS1, fibronectin 1, and ITGB4 are all DEGs in PI3K-Akt signalling pathway at 21 d, and the expression profiles of COL6A3 suggested that it play an important role in pregnancy, and miscarriage was initiated by its abnormal expression [25]. In particular, MMP14, VCAM1, and MMP9 of DEGs in tumour necrosis factor signalling pathway further emphasized these genes during pregnancy, and MMP14 is important for maintenance of pregnancy in cattle [26]; VCAM1 is involved in the bovine conceptus adhesion to the uterine endometrium [27]. In addition, the Hippo signalling pathway play an important role in the preimplantation mouse development [28], and miscarriage was caused by the dysfunction of the Hippo signalling pathway.

In conclusion, our results identified genes and pathways crucial to pregnancy and miscarriage in cows.

## Figures and Tables

**Table 1:** Primers for real time polymerase chain reaction

Gene name	Primers
*LILRA4*	Forward: GCCAGGTTCTCCATCCAGTA
	Reverse: CTCCTTCCTGTCACCACCAG
*CDH2*	Forward: CCTCTGGATCGTGAGCTGAT
	Reverse: CCCTCAGGAACTGTCCCATT
*MMP14*	Forward: GCCTTGAGCATTCCAACGAT
	Reverse: GCACGAAGTTCTCTGTGTCC
*VCAM1*	Forward: TACCAGCTCCACGGATTCTC
	Reverse: CCCAGAATCTTCTGCCCTCA
*LOC100139209*	Forward: TCGTCACCTCTGGACAGAAC
	Reverse: TTGGACAGAGCGAATCTGGT
*EEF1A1*	Forward: AACTCGCCCAACTGACAAAC
	Reverse: CCCACAGGGACAGTACCAAT
*PRSS2*	Forward: CTCCAGGGCATTGTGTCTTG
	Reverse: TCCACGTAGTTGCAGACCTT
*BoLA-DQA*	Forward: AGGTTCCAGAGGTGACTGTG
	Reverse: ACGTGACAGATGAGGGTGTT
*BOLA-DQB*	Forward: TGCTCGGTGACGGATTTCTA
	Reverse: CTCCAGCATCACGAGGATCT
*GAPDH*	Forward: CATGACCACTTTGGCATCGT
	Reverse: CCATCCACAGTCTTCTGGGT

*LILRA4*, leukocyte immunoglobulin like receptor A4; *CDH2*, cadherin 2; *MMP14*, matrix metallopeptidase 14; *VCAM1*, vascular cell adhesion molecule 1; *LOC100139209*, leukocyte immunoglobulin-like receptor subfamily A member 6; *EEF1A1*, eukaryotic translation elongation factor 1 alpha 1; *PRSS2*, serine protease 2; *BoLA-DQA*, *Bos taurus* MHC class II antigen A; *GAPDH*, glyceraldehyde-3-phosphate dehydrogenase.
